# Highlighting the CORD‐SaFe Trial From Melbourne—Progress Toward Cellular Therapy in Extremely Preterm Newborns

**DOI:** 10.1002/pdi3.70021

**Published:** 2025-12-12

**Authors:** Aditya Bhatt, Somashekhar Nimbalkar, Dipen Patel, Reshma Pujara

**Affiliations:** ^1^ Department of Neonatology Pramukhswami Medical College Bhaikaka University—Karamsad Anand Gujarat India

## 1 Introduction

Preterm brain injury remains a significant challenge for extremely preterm infants (< 28 weeks gestation), often leading to long‐term neurodevelopmental impairments. Despite advancements in antenatal care, neonatal therapeutics for the prevention or treatment of brain injuries are limited [1]. Umbilical cord blood (UCB) has emerged as a promising source of stem cells, offering neuroprotective effects via immune‐modulating mechanisms observed in preclinical models [2, 3]. Although autologous UCB‐derived therapies have shown safety and efficacy in term and moderately preterm neonates, their feasibility and safety in highly preterm infants had not been studied. The CORD‐SaFe study, published earlier in 2025 in *eBioMedicine*, aimed to evaluate the feasibility of collecting, processing, and administering autologous UCB, as well as the safety of intravenous reinfusion of UCB‐derived cells in this high‐risk population [4]. Here, we aim to discuss an essential original study published in *The Lancet* [4]. We conducted no separate formal analysis apart from that published by Zhou et al.

## 2 Methodology

The CORD‐SaFE trial was a single‐center, open‐label, single‐arm phase I clinical trial conducted at Monash Medical Center and Monash Children's Hospital in Melbourne, Australia. Eligible participants included extremely preterm infants (< 28 weeks gestation) without major congenital malformations or severe brain injuries. UCB was collected at birth using sterile techniques and processed to isolate mononuclear cells (MNCs). Cryopreserved cells were reinfused intravenously during the second postnatal week at doses of 25–50 million MNCs/kg. Feasibility outcomes included sufficient UCB volume (> 7 mL) and adequate cell counts (> 25 × 10^6^ total nucleated cells/kg). Safety outcomes focused on adverse events related to cell infusion. Exploratory analyses included cytokine profiling before and after infusion.

**TABLE 1:** Demographic data for infants receiving autologous UCBC infusion, and contemporaneous cohort not receiving UCBC infusion.

Demographics	Infused infants (*N* = 23)	Contemporaneous cohort (*N* = 93)
Perinatal		
Maternal age (years)	35 (30–37)	32 (29–35)
Maternal body mass index	28 (23–32)	27 (24–33)
Pregnancy induced hypertension	4/23 (17.3%)	11/93 (11.8%)
Gestational diabetes	7/23 (30.4%)	11/93 (11.8%)
Chorioamnionitis	6/23 (26.0%)	6/93 (6.4%)
Prolonged rupture of membranes > 18 h	12/23 (52.1%)	35/93 (37.6%)
Antepartum hemorrhage	3/23 (13.0%)	27/93 (29.0%)
Fetal growth restriction	8/23 (34.7%)	16/93 (17.2%)
Antenatal steroids (complete course)	22/23 (95.6%)	60/93 (64.5%)
Magnesium sulfate at delivery (any)	23/23 (100%)	79/93 (84.9%)
Caesarean section	19/23 (82.6%)	51/93 (54.8%)
Deferred cord clamping, at least 60 s	16/23 (69.5%)	66/93 (70.9%)
Intubation at birth	6/23 (26.0%)	27/93 (29.0%)
Surfactant at birth	5/23 (21.7%)	15/93 (16.1%)
Neonatal		
Gestational age at birth (completed weeks)	26 (25–27)	26 (24–27)
Birth weight (grams)	750 (650–946)	769 (660–1017)
Male sex	17/23 (73.9%)	39/93 (41.9%)
Apgar score at 5 min	8 (6–9)	8 (7, 8)
IVH grade 1–2 (pre‐infusion)	13/22 (59.0%)	48/93 (51.6%)
Patent ductus arteriosus treated (pre‐infusion)	13/22 (59.0%)	42/93 (45.1%)

*Note:* UCBCs are autologous mononuclear cells isolated from an infant’s own cord blood and processed under GMP conditions for intravenous infusion. Data are expressed as median (inter‐quartile range) or *n* (%) [4].

Abbreviations: GMP, good manufacturing practice; IVH, intraventricular hemorrhage; UCBC, umbilical cord blood‐derived cells.

## 3 Results

**FIGURE 1:**
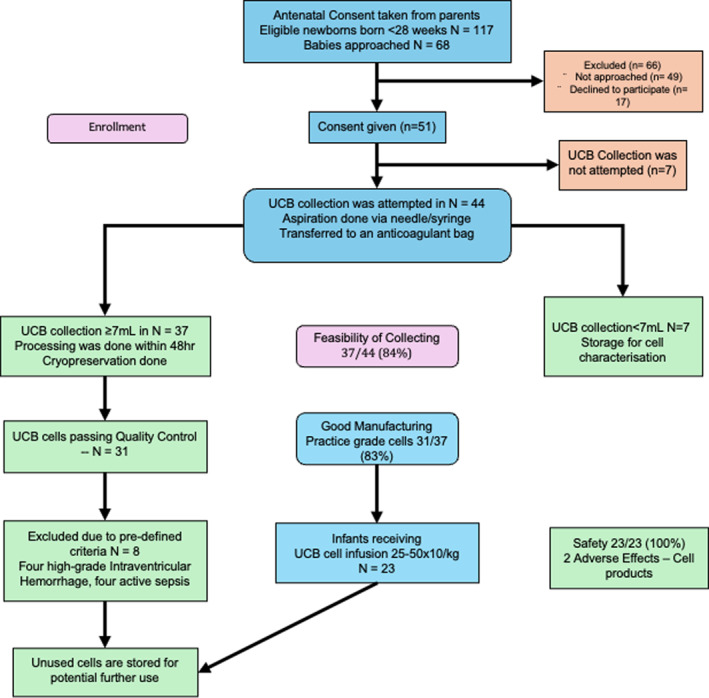
Consort diagram depicting CORD‐SaFe study recruitment from May 2021 to November 2023.

Of 117 eligible infants, 51 consented, and UCB collection was attempted in 44. Adequate UCB volume and cell extraction were successful in 37 cases (84.1%). Good manufacturing practice‐grade umbilical cord blood‐derived cells (UCBCs) were available in 31 (70.5%) of those attempts (Figure 1). Ultimately, *23 extremely preterm infants* received autologous UCBC infusion. The median gestational age was 26 weeks, and the median cell dose infused was 42.3 million mononuclear cells per kilogram (Table 1).

No serious adverse events were directly related to the infusion. Vital signs remained stable before, during, and after the infusion. There was one death, unrelated to the treatment, due to a bloodstream infection. Two cases had post‐production microbial contamination; however, blood cultures from those infants were negative and both remained stable.

Feasibility was further supported by the relatively high cell counts and adequate processing quality, even in the context of deferred cord clamping, which was performed in 69.5% of cases. Although some UCB units were excluded due to contamination or blood typing issues, the overall success rate was considered high enough to justify advancing to the next trial phase.

Exploratory biomarker changes observed in this study require validation in future controlled studies with appropriate comparison groups. These preliminary findings cannot be interpreted as evidence of therapeutic efficacy and should be considered hypothesis‐generating for future research. A comparison with a contemporaneous cohort (*n* = 93) which did not receive UCBC therapy showed no significant differences in major neonatal morbidities, although the trial was not powered for efficacy comparisons.

## 4 Conclusion

The CORD SaFe trial has established that UCB collection and reinfusion are feasible for approximately 70% of extremely preterm infants. The therapy was well‐tolerated, with no serious adverse events directly attributable to the UCBC infusion. The findings by Zhou et al. provide a foundation for further studies exploring the efficacy of autologous UCB‐derived cell therapy in reducing preterm brain injury and improving neurodevelopmental outcomes [4].

Future research will include long‐term follow‐up assessments to evaluate developmental outcomes and refine therapeutic protocols for broader clinical application. These results support progressing to more extensive controlled trials to assess *efficacy*. The next step, the *CORD‐CELL trial*, is already underway to further evaluate outcomes, including long‐termneurodevelopmental effects [5].

## Author Contributions

**Aditya Bhatt:** conceptualization, formal analysis, writing – original draft. **Somashekhar Nimbalkar:** conceptualization, formal analysis, writing – review and editing, supervision, visualization, validation. **Dipen Patel:** visualization, writing – review and editing. **Reshma Pujara:** validation, writing – review and editing.

## Conflicts of Interest

The authors declare no conflicts of interest.

## Data Availability

The data that support the findings of this study are available from the corresponding author upon reasonable request.
